# The hierarchical structure of error-related negativities elicited from affective and social stimuli and their relations to personality traits

**DOI:** 10.1017/pen.2020.15

**Published:** 2021-01-05

**Authors:** T. Suzuki, K. D. Novak, B. Ait Oumeziane, D. Foti, D. B. Samuel

**Affiliations:** 1 Department of Psychological Sciences, Purdue University, West Lafayette, IN, USA; 2 Department of Psychiatry, University of Michigan, Ann Arbor, MI, USA; 3 VA Boston Healthcare System, Boston, MA, USA

**Keywords:** Error-related negativity, Five-factor model, Personality inventory for DSM-5, Psychophysiology, Measurement

## Abstract

Psychophysiological measures have become increasingly accessible to researchers and many have properties that indicate their use as individual difference indicators. For example, the error-related negativity (ERN), an event-related potential (ERP) thought to reflect error-monitoring processes, has been related to individual differences, such as Neuroticism and Conscientiousness traits. Although various tasks have been used to elicit the ERN, only a few studies have investigated its variability across tasks when examining the relations between the ERN and personality traits. In this project, we examined the relations of the ERN elicited from four variants of the Flanker task (Arrow, Social, Unpleasant, and Pleasant) that were created to maximize the differences in their relevance to personality traits. A sample of 93 participants with a history of treatment for psychopathology completed the four tasks as well as self-report measures of the general and maladaptive five-factor model (FFM) traits. Confirmatory factor analyses (CFAs) of ERN amplitudes indicated that three of the four tasks (Arrow, Social, and Unpleasant) were unidimensional. Another set of CFAs indicated that a general factor underlies the ERN elicited from all tasks as well as unique task-specific variances. The correlations of estimated latent ERN scores and personality traits did not reflect the hypothesized correlation patterns. Variability across tasks and the hierarchical model of the ERN may aid in understanding psychopathology dimensions and in informing future endeavors integrating the psychophysiological methods into the study of personality. Recommendations for future research on psychophysiological indicators as individual differences are discussed.

Methods of assessing individual differences in physiological and neurological processes are becoming increasingly available to personality and psychopathology researchers (DeYoung et al., 2010; Hyatt et al., 2019; Luu, Collins, & Tucker, 2000; Olvet & Hajcak, 2008). Though personality research has developed primarily using questionnaires, the recent interest in incorporating other methods to refine personality theories has increased substantially (Allen & DeYoung, 2017; Hyatt et al., 2019) as personality is integrated into our understanding of psychopathology dimensions. For example, the Research Domain Criteria (RDoC) proposed by the National Institute of Mental Health explicitly encourages the integration of multiple methods, including physiological methods and questionnaires (e.g., Insel et al., 2010). As the recent founding of this journal (*Personality Neuroscience*) attests, this is an exciting period where the long history of utilizing multi-method approaches in understanding individual differences (Campbell & Fiske, 1959) is leveraging neuroscience methods.

Among the physiological methods available, the event-related potential (ERP) technique is a promising marker of brain activity that can be integrated with personality and psychopathology research. The ERPs are electroencephalogram (EEG) patterns time-locked to events, such as presentation of stimuli or actions taken by participants (Luck, 2014). The main practical advantage of ERPs is its substantially lower cost of data collection compared to other measures of brain activity, such as functional magnetic resonance imaging (fMRI). This facilitates the collection of sample sizes needed for the correlational analyses often used in personality research.

The error-related negativity (ERN; Falkenstein, Hohnsbein, Hoormann, & Blanke, 1991; Gehring, Goss, Coles, Meyer, & Donchin, 1993) is an ERP that has well-established properties and is suitable for an in-depth investigation of individual differences. The ERN is one of the most studied ERPs and is elicited by various speeded reaction tasks, including the Flanker task, Stroop task, and Go/No-Go task (Riesel, Weinberg, Endrass, Meyer, & Hajcak, 2013). The ERN refers to a negative deflection in the ERP waveform that occurs approximately 50–100 ms after the commission of an error. It is thought to reflect preconscious automatic error detection stemming from continuous performance monitoring. The ERN has a frontocentral topography and likely originates from the anterior cingulate cortex, and possibly the pre-supplementary motor area (Dehaene, Posner, & Tucker, 1994; Holroyd & Coles, 2002; Iannaccone et al., 2015). The ERN also has promising properties as an individual difference indicator. For example, the ERN has high internal consistency, such as good split-half reliability (approximately .80; Foti, Kotov, & Hajcak, 2013), and is known to have trait-like properties, including high test–retest reliability (approximately .70 over 2-week and 2-year periods; Olvet & Hajcak, 2009; Weinberg & Hajcak, 2011), making it a promising candidate for examining its relations with other measures of personality and psychopathology.

Various theories have been proposed to explain the exact mechanisms and functions of the ERN (for a review, see e.g., Olvet & Hajcak, 2008). Examples of proposed theories include the mismatch theory, which posits that the ERN is the result of a discrepancy in the expected result (e.g., correct) and the actual result when the response is made (e.g., error; Coles, Scheffers, & Holroyd, 2001), and conflict monitoring theory, which posits that the ERN is the result of continued monitoring of the conflicts (e.g., left-pointing and right-pointing arrows on the same screen) during the response process (Yeung, Botvinick, & Cohen, 2004). Individual differences in ERN amplitudes could reflect one of these performance monitoring mechanisms or other cognitive processes. However, these theories do not explain the effects of motivation on the ERN, such as the modulating effect of monetary incentives on ERN amplitudes (Hajcak, Moser, Yeung, & Simons, 2005; Olvet & Hajcak, 2008). In order to consolidate past cognitive and individual differences findings, the ERN has recently been theorized to reflect defensive reactivity when making an error, which motivates and facilitates adjustments in behavior to maintain effective performance (Weinberg, Riesel, & Hajcak, 2011). This conceptualization of the ERN, as reflecting individual differences in motivation to reduce making errors, could facilitate linking the ERN to the broader personality literature.

The five-factor model (FFM; McCrae & Costa, 2010) is an omnibus model of general personality that has provided a valuable framework for integrating findings across disparate fields, including health psychology (e.g., Marshall, Wortman, Vickers, Kusulas, & Hervig, 1994), workplace performance (Barrick & Mount, 1991), and psychopathology (Samuel & Widiger, 2008; Widiger et al., 2019). In this same way, it is an ideal framework for contextualizing the ERN. The FFM consists of five broad domains: Neuroticism, Extraversion, Openness to Experience, Agreeableness, and Conscientiousness. Neuroticism is characterized by the tendency to experience negative affect; Extraversion is characterized by gregariousness and a tendency to experience positive emotions; Openness to Experience is characterized by a tendency to enjoy new experiences and ideas; Agreeableness is characterized by a tendency to be friendly and altruistic; and Conscientiousness is characterized by a tendency to be organized and disciplined. These domains are considered bipolar, in that they encompass the traits as well as their conceptual opposites (e.g., Introversion is the opposite of Extraversion). The FFM is also hierarchical in that each of these five domains is further differentiated into lower order traits, referred to as facets. One predominant model of the FFM includes six facets beneath each domain that provide a more nuanced description of personality (Costa & McCrae, 1995).

The FFM has been useful as a framework for characterizing psychopathology (e.g., Widiger & Costa, 2012). Decades of research show that the categorical conceptualization of personality disorder (PD) is problematic (Clark, 2007) and that PD categories are better understood as collections of extreme, maladaptive variants of FFM traits (Widiger & Trull, 2007). Informed by such research, the Diagnostic and Statistical Manual of Mental Disorders, Fifth Edition (DSM-5; American Psychiatric Association, 2013), included an alternative model of personality disorder (AMPD) specified in part by dimensional traits. The AMPD trait model, which is operationalized by a measure called the Personality Inventory for DSM-5 (PID-5; Krueger, Derringer, Markon, Watson, & Skodol, 2012), includes five domains that are highly similar to those on measures of the FFM. These domains are Negative Affectivity (similar to Neuroticism), Detachment (inverse of Extraversion), Psychoticism (similar to Openness to Experience), Antagonism (inverse of Agreeableness), and Disinhibition (inverse of Conscientiousness). The PID-5 domains are also broken down into 25 lower order facets. Statistical analyses, such as latent construct analyses (e.g., Gore & Widiger, 2013) and nomological network similarities (Suzuki, Griffin, & Samuel, 2016), indicate that four out of the five PID-5 domains show high convergence with traditional FFM measures. The fifth domains of PID-5 Psychoticism and FFM Openness to Experience also show overlap, but this is more nuanced (Chmielewski, Bagby, Markon, Ring, & Ryder, 2014; DeYoung, Grazioplene, & Peterson, 2012; Moorman & Samuel, 2018). Therefore, the FFM provides a useful framework to investigate individual difference dimensions that range from general to maladaptive.

Several studies have examined the relations between the ERN and personality traits, including the FFM traits. Individuals with larger (more negative) ERNs are often higher on Neuroticism-related traits (e.g., Foti, Kotov, Bromet, & Hajcak, 2012; Hajcak, McDonald, & Simons, 2004) and Conscientiousness-related traits (e.g., Potts, George, Martin, & Barratt, 2006; Stahl, Acharki, Kresimon, Völler, & Gibbons, 2015). Relatedly, the ERN has been linked to various forms of psychopathology, as well. For example, ERN amplitudes have been found to be enhanced among individuals with obsessive-compulsive disorders (Riesel, 2019) and blunted in individuals with externalizing disorders (Hall, Bernat, & Patrick, 2007). Conceptualizing the ERN as reflecting individual differences in defensive reactivity and motivation, ERN amplitudes are likely modulated by the value and context of the errors (Weinberg et al., 2011). Thus, individual differences in the ERN likely relate to these dimensions because they are characterized by reactions to negative outcomes (Neuroticism) and by the motivation (or lack thereof) to limit mistakes (Conscientiousness).

We recently examined the relations between the ERN and the FFM domains (Suzuki, Hill, Ait Oumeziane, Foti, & Samuel, 2018), as assessed through the brief Five-Factor Model Rating Form (Mullins-Sweatt, Jamerson, Samuel, Olson, & Widiger, 2006). In a sample of approximately 160 undergraduate participants, the correlations between the ERN and personality traits were small, with the largest correlation being .16 with Openness to Experience. Neuroticism and Extraversion also correlated at .11 while the other two domains correlated at less than .10 with the ERN. All correlations were positive, indicating blunted (smaller) ERN with higher trait levels. These findings were contrary to our expectations and add to the inconsistent relations found between the ERN and personality traits across studies. This indicated the need to examine assumptions made when investigating relations between the ERN and personality traits.

A key assumption currently made about the ERN is that it is a unitary construct regardless of the task used to elicit it (i.e., the ERN is invariant across tasks). A close examination of this point may be crucial for understanding how the ERN, as elicited from various tasks, relates to other markers of individual differences. It is notable then, that Riesel and colleagues (2013) have shown that ERN amplitudes elicited by three common tasks (Flanker, Stroop, and Go/No-Go tasks) correlated between .64 and .76. This suggests that, although there may be a general variance of ERN that is shared across tasks, there is also task-specific variance. The specific variance across different tasks hold the potential to examine defensive reactivity (i.e., ERN) in different contexts. Further, the ERN elicited by different tasks might form a hierarchical structure, like personality traits, with the General ERN reflecting a domain-level defensive reactivity and the task-specific ERN reflecting facet-level defense reactivity to specific aspects of the tasks. As an illustration, Munro and colleagues (2007) used two Flanker tasks, one with alphabetical characters as stimuli and the other with fearful and angry facial pictures as stimuli. They found that individuals with higher scores on psychopathy had a blunted ERN in the emotional face Flanker task, but not the alphabet Flanker task. This comports with the theory of task-specific variance because psychopathy is conceptualized by interpersonal aggression (e.g., Miller, Lyman, Widiger, & Leukefeld, 2001) making the emotional faces less salient than letters.

Extending this work regarding task effects on the ERN, the current study elicited ERN amplitudes from four variants of the Flanker task to arbitrate the unitary assumption of the ERN (i.e., the task does not matter) and the characteristics of the task-specific variances. First, the ERN was elicited using the commonly used Arrow Flanker task, in order to anchor with previous research. Arrow symbols have no direct emotional or social relevance and, therefore, are hypothesized to be neutral with regard to the personality and reflect a pure version of defensive reactivity. Based on past research findings, we hypothesized that the ERN elicited from the Arrow task would show enhanced amplitudes among individuals higher on Neuroticism and Conscientiousness.

Three more tasks were chosen to elicit ERN amplitudes with more personality-relevant stimuli. These tasks were chosen (1) to maximize the differences across tasks and (2) to capitalize on the differences among the FFM domains. First, a Flanker task with facial stimuli was used to elicit ERN with a more “social” flavor. We modified the task used by Munro and colleagues (2007) to separate the “social” and the “emotional” aspects of the face stimuli they used. These stimuli were chosen based on the working hypothesis that the ERN elicited using faces would reflect individual differences in how much individuals “cared” about the errors made in social contexts. Specifically, we expected that ERN amplitudes elicited from this task would be more enhanced among individuals higher in Extraversion and Agreeableness, given these are interpersonal traits (McCrae & Costa, 1989) and have well-established relations with social outcomes, such as peer acceptance and quality of interpersonal relationships (Ozer & Benet-Martínez, 2006).

The next task we employed was a Flanker task with unpleasant (i.e., negatively valenced) pictures from the International Affective Picture System (IAPS; Lang, Bradley, & Cuthbert, 2005). We chose these stimuli based on the working hypothesis that unpleasant stimuli will be most salient to individuals who are more prone to negative affect. We hypothesized that ERN amplitudes elicited by the Unpleasant task would be most enhanced among individuals higher on Neuroticism trait, given Neuroticism is characterized by the tendency to experience negative affect and closely related to depression and anxiety (McCrae & Costa, 2010).

Our fourth and final Flanker task used pleasant (i.e., positively valenced) pictures from the IAPS. Given that Extraversion is conceptualized as the tendency to experience positive affect in general (McCrae & Costa, 2010), we hypothesized that pleasant stimuli would modulate the ERN such that amplitudes will be more enhanced among individuals higher on Extraversion.

A previous manuscript using the same sample and tasks has confirmed the successful elicitation of the ERN from all four tasks and examined various *within*-subject contrasts on ERP and behavioral measures of this data (Suzuki, Ait Oumeziane, Novak, Samuel, & Foti, 2020). It also examined the correlations of the grand average ERN amplitudes (a traditional single grand average of the ERN using all trials) across tasks that ranged from .42 (the Arrow and Pleasant tasks) to .69 (Unpleasant and Pleasant tasks), indicating potential task-specific variances. The previous manuscript focused on the *within*-subjects effects, such as the elicitation of the ERN and Flanker effects, and the only *between*-subject analysis conducted was the correlations across tasks using the observed grand average ERN amplitudes. The present manuscript focuses on *between*-subject correlational analyses and the latent ERN amplitudes as described below.

The ERN amplitudes elicited from the four tasks were first subjected to confirmatory factor analyses (CFAs) separately to test the unidimensionality of the ERN from each task. This was to test the assumption made when calculating mean ERN amplitudes using all trials, as traditionally done, and all CFAs were expected to fit adequately. Then, scores from all tasks were examined within one model with all trials (operationalized as units in this manuscript; explained in the Methods section) directly loading onto a single latent construct (e.g., the General ERN) to assess the assumption of the ERN as a unitary construct. Given this would indicate a single latent factor underlying all trials (units), this model was not expected to fit adequately. Rather, recent evidence suggests variation in ERN amplitudes elicited across tasks. Thus, we hypothesized a second-order model with the task-specific latent constructs between the general latent construct and trials (units), to improve fit appreciably. Next, to capture their nomological networks, the estimated latent ERN factor scores were correlated with estimated latent FFM general and maladaptive personality trait scores to examine the relations of the general and task-specific ERN scores to personality. We hypothesized that the task-specific ERN amplitudes would correlate most highly with specific personality traits, as outlined above. These predictions were made at the time the tasks were chosen and modified to test the specific hypotheses. For example, we developed the Social task as we thought that the ERN scores elicited from this task would be most relevant to Extraversion and Agreeableness domains. Based on past research showing that the ERN elicited by various Flanker tasks is associated with Neuroticism and Conscientiousness, we expected the General ERN to be correlated highly with Neuroticism and Conscientiousness. All ERN and trait relations were examined simultaneously to provide a multi-trait multi-method matrix within which to contextualize the hypotheses and results. Finally, the personality profile similarities of the ERN scores were explored as another approach to quantify similarities and differences of tasks. These relations were examined within a clinical sample of undergraduate students with a history of psychological treatment to maximize the variability in the ERN and personality traits within an undergraduate sample.

## 1. Method

### 1.1 Participants

Ninety-nine undergraduate students with a lifetime history of psychotherapy or pharmacotherapy for mental illness participated in this study. Lifetime history was assessed broadly through two prescreening questions administered at the beginning of each semester (e.g., “Have you ever been treated with psychotherapy for psychiatric health care or an emotional or mental illness?”) and participants received course credit for their participation in this study. Data from six participants were excluded for the following reasons: one participant did not complete the entire set of laboratory tasks due to technical difficulties, one participant had excessive artifacts (i.e., noise) in their ERP data, and four participants did not correctly answer at least half of the validity items embedded in the self-report questionnaires (four out of six items). This resulted in a final sample of 93 participants that was 59% Female with a mean age of 19.2 years (SD = 1.00). In terms of race/ethnicity the sample was 78% White, 14% Asian, 1% Black or African American, 1% American Indian or Alaskan Native, while 5% Multiracial and 4% indicated Hispanic or Latinx. Further, task data from some participants were not excluded: One participant did not complete the correct Arrow task, three participants had less than 70% accuracy on the Pleasant task, and data appropriate for trial-level analyses could not be extracted from four-task data (one Arrow, two Unpleasant, and one Pleasant). For these participants, data from the other tasks were included. This study was approved by Purdue University’s Institutional Review Board and all participants provided informed consent to participate in this study.

### 1.2 Laboratory tasks

#### 1.2.1 Arrow task

The Arrow Flanker task (Eriksen & Eriksen, 1974) is a task commonly used to elicit the ERN (Endrass et al., 2010; Olvet & Hajcak, 2009). Five arrowheads were presented in the center of the screen on each trial. Participants indicated the direction of the center arrowhead (i.e., left or right) by clicking buttons on a mouse. There were four equiprobable stimuli, which consisted of two congruent (e.g., <<<<<) and two incongruent (e.g., <<><<) stimuli. The stimuli were randomized without replacement (i.e., each stimulus presented 75 times). The intertrial intervals (ITIs) varied randomly from 2500 to 3000 ms and a fixation cross was presented between trials. Participants completed 10 blocks of 30 trials each, for a total of 300 trials. The presentation time of stimuli was initially set to 200 ms and was adjusted after each block (including the practice block) to maintain accuracy between 75% and 90% of the trials. Specifically, presentation times were lengthened by 20 ms if accuracy was below 75% and shortened by 20 ms if accuracy was above 90%. Participants were also given feedback between blocks (i.e., after 30 trials) to respond faster if their performance was above 90% accuracy and to respond more accurately if their performance was below 75%.

#### 1.2.2 Social task

Pictures of faces from the NimStim Face Stimuli Set (Tottenham et al., 2009) with neutral expressions were used to elicit the ERN within a social context. The procedure was generally the same as the Arrow task. Three gray-scaled face stimuli were presented in the center of the screen in each trial. Participants’ task was to identify the orientation of the center picture. There were four equiprobable trial types, which consisted of two congruent (e.g., upright-upright-upright faces of the same actor) and two incongruent (e.g., inverted-upright-inverted faces of the same actor). The stimuli were randomized without replacement (i.e., each stimulus presented 75 times), the ITIs varied randomly from 3500 to 4000 ms (longer than Arrow task), and a fixation cross was presented between trials. Participants completed 10 blocks of 30 trials each. The presentation time of stimuli was initially set to 400 ms to accommodate the increased stimuli complexity as was done by Munro and colleagues (2007). The presentation time was adjusted based on participant performance. Specifically, presentation times were lengthened by 40 ms if accuracy was below 75% and shortened by 40 ms if accuracy was above 90%. Participants were given feedback between blocks based on their performance (e.g., to respond faster if performance above 90% accuracy).

#### 1.2.3 Unpleasant task

Pictures with unpleasant images (e.g., bodily mutilation, threatening pictures) and neutral images (e.g., household items) were drawn from the IAPS (Lang et al., 2005). The general procedure was the same as the Social task and the only differences were the stimuli and instruction. Three gray-scaled stimuli were presented in the center of the screen in each trial. Participants’ task was to identify the valence of the center picture. There were four equiprobable trial types, which consisted of two congruent (e.g., unpleasant-unpleasant-unpleasant images) and two incongruent (e.g., neutral-unpleasant-neutral images). Within a trial, the same picture was used for the same valence (e.g., pictures of aggressive dogs on both sides of a picture of a lamp in an unpleasant-neutral-unpleasant trial). The same randomization, ITI, fixation cross presentation, number of trials, presentation time, presentation time adjustment, and feedback procedures as the Social task were used.

#### 1.2.4 Pleasant task

Pictures with pleasant images (e.g., kittens, erotic) and neutral images were drawn from the IAPS (Lang et al., 2005). The general procedure was the same as the Unpleasant task and the only difference was the stimuli and instruction. There were four equiprobable trial types, which consisted of two congruent (e.g., pleasant-pleasant-pleasant images) and two incongruent (e.g., neutral-pleasant-neutral images). Within a trial, the same picture was used for the same valence (e.g., erotic pictures on both sides of a picture of a lamp in a pleasant-neutral-pleasant trial). The same randomization, ITI, fixation cross presentation, number of trials, presentation time, presentation time adjustment, and feedback procedures as the Social and Unpleasant tasks were used.

#### 1.2.5 Task order

All participants completed the Arrow Flanker task first and the remaining three tasks were administered in a randomized order. This ordering was determined to avoid any unintended effects from the new tasks into the Arrow Flanker task that has been well established in the literature. The other three tasks were not as well established and the ordering was randomized to control for possible order effects.

### 1.3 ERP data recording and processing

**Figure 1.**
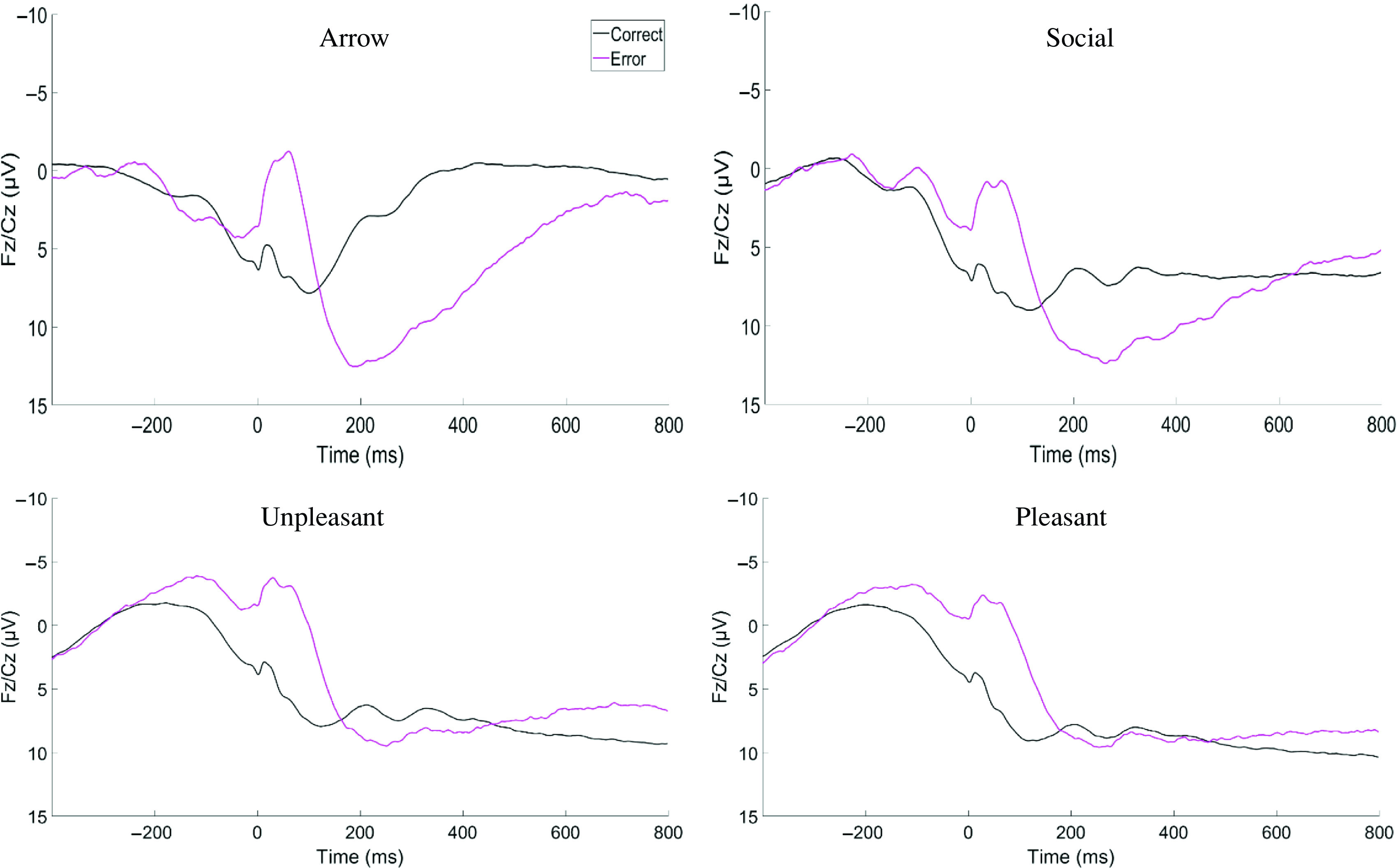
The event-related potential amplitudes of each task (red line indicates the ERP when an error is made and the black line indicates the ERP when a correct response is made).

While participants performed the Flanker tasks, the continuous EEG signal was recorded from 32 Ag/AgCl active scalp electrodes using the actiCAP and actiCHamp system (Brain Products). A 24-bit resolution with a sampling rate of 500 Hz was used to digitize the signals. Electrode impedances were maintained below 30 kOhm. Brain Vision Analyzer software (Brain Products) was used to perform offline analyses. The averaged mastoid (TP9/TP10) was used to re-reference all data. The continuous data were band-pass filtered (from 0.1 to 30 Hz). Then, ERPs were derived by segmenting the signals at −400 to 800 ms around the behavioral responses. To correct for eye movements and blinks, a regression-based method was used (Gratton, Coles, & Donchin, 1983). Artifact rejection was conducted at the individual channel level using a semi-automated procedure (rejecting a step of 50 µV, >200 µV difference in intervals of 200 ms, and <.5 µV difference in intervals of 100 ms, as well as visual inspection). The ERN (error trials minus correct trials) was expected to be captured in the frontal-central medial electrodes (i.e., Fz and Cz) and immediately following a response for 100 ms. However, to ensure consistency in the electrodes and time window across tasks, the *within*-subjects contrast of the error and correct ERP waveforms averaged across participants (Figure 1; blinded to any *between*-subjects effect) were visually inspected to determine the locations and time window that maximized the ERN amplitudes across tasks. ERPs were scored as the mean activity at Fz and Cz electrodes between 10 and 110 ms on the error and correct trials with a baseline correction using a −400 to −200 ms interval. Once the electrodes and the time window were determined, trial-level data were extracted from each participant.

### 1.4 Self-report measures

#### 1.4.1 General personality

The International Personality Item Pool-NEO (IPIP-NEO; Goldberg, 1999; Maples, Guan, Carter, & Miller, 2014) was used to assess general personality traits. It is a 120-item measure that assesses the five broad domains (Neuroticism, Extraversion, Openness to Experience, Agreeableness, and Conscientiousness) as well as the 30 facets. Each facet is assessed by four items.

#### 1.4.2 Maladaptive personality

A shortened version of the Personality Inventory for DSM-5 (PID-5; Krueger et al., 2012; Maples et al., 2015) was used to assess maladaptive personality traits. It assesses the five broad domains (Negative Affectivity, Detachment, Psychoticism, Antagonism, and Disinhibition) as well as the 25 finer facets that are assessed by four items each. Negative Affectivity has seven facets, Detachment was measured by five facets, Psychoticism was measured by three facets, Antagonism was measured by five facets, and Disinhibition was measured by five facets. Please refer to Maples et al. (2015) for the exact domain-facet specifications.

## 2. Analytic plan

First, the data from Fz and Cz electrodes were averaged to calculate trial-level ERP scores. The trial-level ERP amplitudes were then screened for any extreme values. This was conducted on data separated by participant, task, and result (correct and error). Specifically, within each participant, trial-level data were separated into the four tasks from which they were elicited (e.g., four 300-trial data for each participant). Within each task, these were further separated into correct and error trials. Thus, for participants with complete data, within-participant outliers were screened in eight separate bins (e.g., Arrow task correct trials, Social task error trials). Then, the means and standard deviations (SDs) of the amplitudes were calculated within these separate data bins for each participant independently. Finally, trials with ERP amplitudes that were below or above three SDs of the mean within each data bin were removed (see Supplemental Table 1 for the characteristics of the trials removed through this procedure).

These data without within-participant outliers in the data bins were sorted back into four task-specific databases to calculate various mean amplitudes. First, ERP trials (e.g., 300 trials for participants with complete data within a task) were divided into five units of 60 trials each, defined by their trial numbers, regardless of the result. Five units were chosen to balance the need to have multiple variables to test unidimensionality within a CFA framework (i.e., more than three) and to retain as many trials within each unit to maximize the likelihood of including an error trial. In addition, the tasks were administered in 10 blocks of 30 trials, making five units a convenient approach. Specifically, Unit 1 consisted of the ERP amplitudes from trials 1 through 60, Unit 2 consisted of the ERP amplitudes from trials 61 through 120, and so on. Therefore, for example, Unit 1 consisted of the average of the ERP amplitudes from the beginning of the task and Unit 5 consisted of the average of the ERP amplitudes from the end of the task (i.e., trials 241 to 300 or blocks 9 and 10). Then, grand average (i.e., ignoring units) and unit-level between-subjects ERP outliers were removed (e.g., below or above three SDs of Unit 1 mean across participants). Finally, for both grand average and unit-level ERPs, the ERN amplitudes were calculated by subtracting the average ERP amplitudes of the correct trials from the ERP amplitudes of the error trials. At least one error needed to be made within a unit (see Supplemental Table 2 for descriptive statistics of the number of errors made in each unit) and ERPs from correct and error trials were needed to calculate the ERN (e.g., Unit 1 correct and error ERPs needed to calculate Unit 1 ERN). Across all participants and tasks, there were a total of 14 units without any error and these units were treated as missing data.

Next, the five ERN units were subjected to CFAs to test the unidimensionality of the ERN within each task (i.e., do the unit ERN amplitudes indicate a latent single task ERN?). To test the unitary construct assumption of the ERN (i.e., do the ERN across tasks reflect a single latent, General ERN?) a model with all 20 units (five from each of the four tasks), loading onto a single latent domain was examined. Next, a second-order CFA model with a General ERN and intermediate latent task constructs were analyzed to test if the tasks had unique variances. Specifically, all four-task latent factors (defined by their units) were specified to load onto a single, General ERN. CFAs were also conducted on personality domains using the facet scores as the data to keep the procedure of estimating latent scores consistent across the ERN and traits. The fittings of the CFAs were judged good if CFI ≥ 0.95, TLI ≥ 0.95, RMSEA ≤ 0.08, and SRMR ≤ 0.08 (Hu & Bentler, 1999; MacCallum, Browne, & Sugawara, 1996). If a task or domain did not indicate a good fit, a good fitting model was identified iteratively by examining every possibility of dropping a unit or a facet to identify a unidimensional task or domain, while dropping as little unit or facet as possible. If multiple models were identified as good fitting during this iterative process, the model with the lower Akaike Information Criterion (AIC) and Bayesian Information Criterion (BIC) was retained for further analyses.

The regression-based estimated scores of the good fitting latent constructs were saved to examine the correlations between ERN amplitudes and personality traits. Given the relatively small sample size and the number of comparisons that were to be made (i.e., 90), effect sizes were used for interpretation rather than statistical significance. The guideline published by Cohen (1992) of *r* ≥ .10 as small effect size, *r* ≥ .30 as medium effect sizes, and *r* ≥ .50 as large effect sizes were used. The personality profile similarities across tasks were computed by correlating the z-transformed correlation between task and personality traits (e.g., the five correlations between latent Arrow ERN and FFM domains were z-transformed and then were correlated with the five z-transformed correlations between latent Social ERN and FFM domains; Suzuki et al., 2018; Westen & Rosenthal, 2003).

All analyses were conducted in R (R Core Team, 2019). Data organization was conducted using reshape2 package (Wickham, 2017), descriptive and correlation analyses were conducted using the psych package (Revelle, 2019), and CFAs and latent score estimations were conducted using the lavaan package (Rosseel, 2012) using robust maximum likelihood estimator and full information maximum likelihood to account for missing data.

## 3. Results

The descriptive statistics of the final ERN data are presented in Supplemental Table 3 and the ERP waveforms are presented in Figure 1. Supplemental Table 4 presents the correlations across units as well as with the grand average. Correlations across units within a task generally ranged from approximately .20–.60. Correlations between individual units and grand averages for that task ranged from approximately .60–.80. Single-factor CFAs of the units within each task indicated that models for three out of the four tasks (Arrow, Social, and Unpleasant tasks) had good fits (see Table 1 for all ERN CFA fit indices). However, the CFA of the Pleasant task indicated a poor fit. By examining five models dropping one unit at a time, dropping either Unit 2 or 4 resulted in good fitting models and AIC and BIC indicated that dropping Unit 4 was the preferred model, which was retained for the rest of the analyses. The factor loadings of these models are presented in Supplemental Table 5. Next, a single-factor CFA with all units loading onto a single domain (i.e., no task-specific latent constructs) indicated poor fit. Finally, the second-order model with the task-specific latent constructs with one overarching general latent ERN indicated a good fit^1^. A figurative representation of the final second-order model is presented in Supplemental Figure 1.

**Table 1.** Fit indices of the error-related negativity task confirmatory factor analyses

Task (Dropped unit)	χ^2^	df	*p*	CFI	TLI	RMSEA	90% CI	SRMR	AIC	BIC
Arrow	1.241	5	.941	1.000	1.098	0.000	[0.000, 0.000]	0.017	2875.606	2913.269
Social	4.138	5	.530	1.000	1.030	0.000	[0.000, 0.126]	0.035	2737.010	2774.999
Unpleasant	4.282	5	.510	1.000	1.024	0.000	[0.000, 0.124]	0.034	2749.581	2787.244
Pleasant	20.848	5	.001	0.779	0.558	0.190	[0.103, 0.286]	0.065	2731.065	2768.225
Pleasant (2)	0.132	2	.936	1.000	1.137	0.000	[0.000, 0.000]	0.009	2203.537	2233.265
Pleasant (4)	0.998	2	.607	1.000	1.105	0.000	[0.000, 0.150]	0.025	2185.526	2215.254
All tasks: Single factor	197.064	152	<.001	0.877	0.861	0.056	[0.031, 0.077]	0.083	10 469.366	10 613.724
All tasks: Second order	147.723	148	.491	1.000	1.001	0.000	[0.000, 0.047]	0.072	10 426.424	10 580.913
Arrow and picture: Second order	159.376	150	.285	0.974	0.971	0.026	[0.000, 0.055]	0.072	10 436.450	10 585.873

χ^2^, chi-square statistic; df, degrees of freedom; *p*, statistical significance; CFI, Comparative fit index; TLI, Tucker–Lewis index; RMSEA, Root mean squares error or approximation; CI, Confidence Interval; SRMR, Standardized root mean square residual; AIC, Akaike Information Criteria; BIC, Bayesian Information Criteria.

The descriptive statistics of the personality data are presented in Supplemental Table 6. The CFAs for the personality domains indicated that only IPIP-NEO Neuroticism domain had a good fit with all original facets. PID-5 Psychoticism was not tested since it only has three facets and is a just-identified model. The remaining eight domains were iteratively tested to find a good fitting model (Table 2). For all domains except IPIP-NEO Agreeableness, PID-5 Detachment, and PID-5 Disinhibition, the model could reach a good fit by dropping only one of the facets. For IPIP-NEO Agreeableness, two facets needed to be dropped, and AIC and BIC indicated dropping facets 3 (Altruism) and 5 (Modesty) was preferred. For PID-5 Detachment, dropping either Anhedonia, Depressivity, or Withdrawal led to good fitting models. AIC and BIC indicated that dropping Withdrawal was the preferred model. For PID-5 Disinhibition, dropping either Distractibility, Impulsivity, or Risk-Taking facets led to good fitting models. AIC and BIC indicated dropping Distractibility facet was the preferred model. The latent scores of these final personality domain models were saved for the subsequent correlation analyses. The correlations across these estimated personality domain scores are presented in Supplemental Table 7.

**Table 2.** Fit indices of the international personality item Pool-NEO and personality inventory for DSM-5 domains

Model	Domain (Dropped facet)	χ^2^	df	*p*	CFI	TLI	RMSEA	90% CI	SRMR	AIC	BIC
IPIP-NEO	N	10.839	9	.287	0.984	0.973	0.047	[0.000, 0.132]	0.042	1265.765	1311.352
	E	17.481	9	.042	0.940	0.900	0.101	[0.027, 0.167]	0.061	1253.562	1299.149
	O	20.050	9	.018	0.844	0.740	0.115	[0.041, 0.187]	0.065	1388.189	1433.776
	A	65.320	9	.000	0.509	0.182	0.259	[0.194, 0.331]	0.125	1117.598	1163.185
	C	29.649	9	.001	0.832	0.720	0.157	[0.097, 0.221]	0.073	1156.593	1202.180
IPIP-NEO facets dropped	E (3)	6.298	5	.278	0.988	0.976	0.053	[0.000, 0.157]	0.037	1041.216	1079.205
	O (4)	5.488	5	.359	0.988	0.977	0.032	[0.000, 0.151]	0.040	1174.877	1212.866
	A (3 and 5)	1.297	2	.523	1.000	1.060	0.000	[0.000, 0.191]	0.027	772.808	803.199
	C(5)	7.319	5	.198	0.975	0.950	0.071	[0.000, 0.168]	0.053	913.345	951.334
PID-5	NA	37.122	14	.001	0.730	0.595	0.133	[0.084, 0.184]	0.089	1355.114	1408.299
	DE	8.711	5	.121	0.959	0.919	0.089	[0.000, 0.174]	0.051	810.560	848.549
	AN	17.575	5	.004	0.876	0.751	0.164	[0.084, 0.253]	0.063	722.227	760.216
	DI	13.818	5	.017	0.859	0.718	0.138	[0.047, 0.234]	0.053	921.968	959.957
PID-5 facets dropped	NA (Restricted affectivity)	11.027	9	.274	0.969	0.948	0.049	[0.000, 0.127]	0.054	1133.006	1178.593
	DE (Withdrawal)	1.629	2	.443	1.000	1.017	0.000	[0.000, 0.173]	0.022	661.604	691.995
	AN (Grandiosity)	1.971	2	.373	1.000	1.001	0.000	[0.000, 0.219]	0.027	609.572	639.963
	DI (Distractibility)	1.246	2	.536	1.000	1.063	0.000	[0.000, 0.174]	0.024	727.743	758.135

χ^2^, chi-square statistic; df, degrees of freedom; *p*, statistical significance; CFI, Comparative fit index; TLI, Tucker–Lewis index; RMSEA, Root mean squares error or approximation; CI, Confidence Interval; SRMR, Standardized root mean square residual; AIC, Akaike Information Criteria; BIC, Bayesian Information Criteria; IPIP-NEO, International Personality Item Pool-NEO; PID-5, Personality Inventory for DSM-5; N, Neuroticism; E, Extraversion; O, Openness to Experience; A, Agreeableness; C, Conscientiousness; NA, Negative Affectivity; DE, Detachment; AN, Antagonism; DI, Disinhibition.

The correlations between latent ERN scores and latent personality scores are presented in Table 3 (see Supplemental Figures 2 and 3 for scatter plots). The latent ERN scores estimated within single-factor CFA were used for each task ERN score and the General ERN score estimated in the second-order CFA was used for the General ERN score. (see Supplemental Table 8 for the same analyses using scores estimated for each task within the second-order CFA model). The ERN is a negative deflection, meaning that enhanced ERN is more negative and blunted ERN is less negative. Therefore, for example, negative correlations between a trait and an ERN amplitude indicate a higher trait level is associated with a larger ERN amplitude. None of the correlations were statistically significant at *p* = .01 threshold. As noted above, we interpreted all relations greater than .10 to be potentially meaningful to examine any emerging relations. Of all the correlations, the largest effect size was *r* = −.23 (the Unpleasant task and IPIP-NEO Agreeableness), indicating generally small correlations across the tasks and domains. The Arrow Task ERN score correlated meaningfully with multiple traits across measures. Specifically, larger ERN (i.e., more negative) amplitudes on the Arrow task was consistently related to higher Agreeableness and to higher Conscientiousness traits with small effect sizes. It was also meaningfully related to higher IPIP-NEO Openness to Experience, but not with PID-5 Psychoticism. Conversely, the Arrow task ERN amplitude was not related to IPIP-NEO Extraversion, but meaningfully related to higher PID-5 Detachment. The Social task ERN correlated at less than .10 with all traits. Larger ERN amplitude on the Unpleasant task is consistently related with small effect sizes in the direction of higher Agreeableness, similar to the Arrow task. At the same time, opposite of the Arrow task, larger ERN elicited from the Unpleasant task was not related to IPIP-NEO Openness, but was meaningfully related to lower PID-5 Psychoticism. The ERN elicited from the Pleasant task meaningfully related to lower Openness to Experience and higher PID-5 Negative Affectivity. Finally, General ERN did not have a meaningful correlation (i.e., less than .10) with any personality traits. Only 19% of expected relations (Arrow task with Conscientiousness and Disinhibition; Unpleasant task with Negative Affectivity) had correlations with at least small effect sizes. In terms of discriminant validity, 74% of the relations not expected to relate were not meaningfully related.

**Table 3.** Correlations of the estimated latent task and general error-related negativity amplitudes from the second-order model with estimated latent international personality item Pool-NEO and personality inventory for DSM-5 domain traits

Model	Domain	Single task CFA				Second-order CFA
		Arrow	Social	Unpleasant	Pleasant	General
IPIP-NEO	Neuroticism	**.10**	.04	−**.09**	−.08	−**.01**
	Extraversion	.04	−**.09**	.03	−**.05**	−.03
	Openness	−.13	−.01	.00	.17	−.02
	Agreeableness	−.15	−**.01**	−.23	−.03	−.09
	Conscientiousness	−**.12**	.00	.02	−.05	−**.05**
PID-5	Negative Affectivity	**.06**	−.04	−**.12**	−.12	−**.08**
	Detachment (−E)	−.18	−**.02**	−.04	**.08**	−.04
	Psychoticism	.02	.00	.15	.15	.08
	Antagonism (−A)	.13	−**.03**	.15	−.01	.06
	Disinhibition (−C)	**.11**	−.06	.07	.03	**.03**

CFA, Confirmatory factor analysis; IPIP-NEO, International Personality Item Pool-NEO; PID-5, Personality Inventory for DSM-5; −E, Opposite of Extraversion; −A, Opposite of Agreeableness; −C, Opposite of Conscientiousness; Bold, Hypothesized relations; Underline, |*r*| > .10.

These correlational patterns were used to calculate personality profile similarities across latent task-specific ERN scores from the single CFA models and the General ERN score from the second-order CFA. The results are presented in Table 4 (see Supplemental Table 9 for the same analyses using scores estimated for each task within the second-order CFA model). This analysis quantifies the rank-order similarity of the correlations between the ERN and domain scores across tasks and can be interpreted as a correlation. There was a range of personality profile similarities across tasks. For example, the IPIP-NEO profiles of the Arrow and Social ERN scores are not similar at all (*r* = −.03), Arrow and Pleasant ERN scores have moderately dissimilar IPIP-NEO profiles (*r* = −.49), and Unpleasant and Pleasant ERN scores have very similar IPIP-NEO profiles (*r* = .68). PID-5 profile similarities also ranged from small to large, indicating personality profile variability across tasks. These PID-5 profile similarities were generally stronger than the IPIP-NEO profile similarities, except Social and Unpleasant tasks reversed in direction (e.g., dissimilar IPIP-NEO profiles, but similar PID-5 profiles).

**Table 4.** Personality profile similarities of estimated latent task-specific and general error-related negativity amplitudes

			Single task CFA			Second-order CFA
			Arrow	Social	Unpleasant	General
Using IPIP-NEO Domains	Single Task CFA	Arrow				**.65**
		Social	−.03			.07
		Unpleasant	.22	−.39		**.68**
		Pleasant	−.49	−.05	.22	.21
Using PID-5 Domains		Arrow				.40
		Social	−.48			.27
		Unpleasant	.39	.27		**.99**
		Pleasant	−.40	**.59**	**.62**	**.64**

CFA, Confirmatory factor analysis; IPIP-NEO, International Personality Item Pool-NEO; PID-5, Personality Inventory for DSM-5; Underline, |*r*| > .30; Bold, |*r*| > .50; Top four rows used correlations with FFM domains to calculate the similarity. Bottom four rows used correlations with PID-5 domains to calculate the similarity.

## 4 Discussion

This manuscript examined the assumptions and the structure of the ERN elicited from multiple tasks as well as their personality trait correlates. The CFAs of ERN amplitudes indicated that the Arrow, Social, and Unpleasant tasks were unidimensional. This supports the historical and continued practice of calculating single grand average means for these tasks. On the other hand, the Pleasant Flanker task required dropping one of the five temporal units to attain unidimensionality. This was despite a successful elicitation of the ERN in the same sample (Suzuki et al., 2020) and suggests that unidimensionality cannot be assumed simply because *within*-subject contrasts are found. Therefore, an explicit examination of the psychometric properties is needed to confidently use summary ERN amplitudes (e.g., grand average) as individual differences indicators. This point is worth emphasizing because relatively few studies have examined the psychometric properties of the ERPs (e.g., Foti et al., 2013; Olvet & Hajcak, 2009).

The unitary construct assumption of the ERN was examined by testing the model with a single latent construct underlying all units, without intermediate task-specific latent constructs. This model did not fit adequately and this result seriously challenges the assumption that the ERN amplitudes elicited from different tasks reflect the same construct. On the other hand, a second-order CFA model with an overarching General ERN factor and intermediate task-specific latent constructs indicated a good fit. This provides evidence that various ERN tasks elicit a common component, but also that tasks are not interchangeable and statistically meaningful task-specific variances exist, as recently speculated (Riesel et al., 2013). Therefore, tasks may have important roles in relating the ERN with individual difference indicators (e.g., personality traits, psychopathology) and explain some inconsistent results in the literature.

To clarify the roles of the tasks and the nomological network of various ERN amplitudes, the task-specific and General ERN amplitudes factors were correlated with all five general and maladaptive FFM domains. These relations were generally small, with the strongest zero-order correlation being *r* = −.23 (between IPIP-NEO Agreeableness and the ERN elicited by the Unpleasant task) and none were statistically significant. This likely reflects the reality of cross-method correlations of the questionnaire and other (e.g., behavioral) measures (e.g., Cyders & Coskunpinar, 2011; Lauriola, Panno, Levin, & Lejuez, 2014) and this is consistent with our previous finding for ERPs (Suzuki, et al., 2018). Further, not only were these correlations small, the patterns of the ERN and personality correlations differed from our hypotheses. Within this data, larger ERN amplitude elicited from the Arrow task related to higher Agreeableness- and Conscientiousness-related traits. Larger ERN is also related to higher IPIP-NEO Openness to Experience. However, these correlations are in the *opposite* direction of our previous work that used a similar setup conducted with the same lab, equipment, and other similarities. Such inconsistency of correlations between self-reported personality and measures of the brain (e.g., structural magnetic resonance imaging) seems to be the reality even with large sample sizes (Valk et al., 2019). We explore possible reasons for why such inconsistency and differences in findings later in this section.

In addition to the commonly examined bivariate relations between the ERN amplitudes and individual difference indicators, we examined the personality profile similarities to gain a different perspective on how the tasks are similar to and different from each other (Westen & Rosenthal, 2003). The personality profile similarities ranged from near zero (e.g., Arrow and Social task ERNs IPIP-NEO profiles) to essentially identical profiles (Unpleasant task and General ERNs profiles on the PID-5). In general, PID-5 profiles for the ERN tasks were more similar than their IPIP-NEO profiles. One interpretation of this finding is that the tasks function very similarly at the maladaptive ends of the dimensions, whereas it may be more differentiated and useful in the general range of personality dimensions. Alternatively, this could simply be a result of the generally higher correlations among the PID-5 domains compared to the IPIP-NEO domains (Supplemental Table 7). However, it is unclear what these patterns indicate and the small sample size (*n* = 93), small correlations, as well as the small number of external correlates (i.e., five domains) limit the interpretability (and likely replicability) of these correlations.

In light of the inconsistencies for ERN and trait relations found between this and our prior study, we start with three important differences between those two projects. First, the personality measures used were different. In the previous study, a much shorter 30-item measure of FFM was used (i.e., FFMRF; Mullins-Sweatt et al., 2006). This study used a longer, 120-item measure that, simply due to length, more reliably assessed the general FFM personality traits. Measure differences also emerged in this manuscript, as well, where PID-5 personality profile similarities of the ERN amplitudes were stronger than the FFM profile similarities. In addition, the CFA of personality domains led to the removal of at least one facet for each IPIP-NEO domain (except Neuroticism), which may have led to latent constructs that were influenced by sample-specific variation. Second, though both studies used undergraduate student samples, the previous study used a general sample while this project used a sample with a history of treatment for mental illness. This sample difference may have important implications, such as potentially increased variances in the ERN and possible nonlinear relations between the ERN and personality traits.

Third, some parameters of the Arrow tasks were different between the two studies. Specifically, the Arrow task used in this manuscript adjusted stimuli presentation time based on participant performance whereas Suzuki and colleagues (2018) did not. Therefore, the Arrow task used in this study likely maintained the difficulty of the task relatively constant across participants while the difficulty varied across participants in the previous study (e.g., the task remained easy for some participants). Since engagement has been found to modulate the ERN (e.g., Luu et al., 2000), this could have elicited ERN amplitudes that were qualitatively different from the previous experiment, yielding different results. Further, the ERN elicited from the Social task did not meaningfully relate to any personality trait. This task was directly modeled after the social component of the task that Munro and colleagues (2007) found to relate to psychopathy, a trait that is a mixture of FFM traits, particularly being low on Agreeableness (Miller et al., 2001). At the same time, the ERN elicited from the Unpleasant task consistently related meaningfully positively (i.e., *r* > .10) with Agreeableness traits. These might indicate that the results obtained by Munro and colleagues (2007) were due to the negative affect component (e.g., anger/sadness) and that the social nature of the stimuli (e.g., faces) did not contribute to the relation. These findings further add to the importance of examining task-specific relations and indicate that task modifications could alter the nature of the ERN.

Another, more general, possible reason for the inconsistent findings is the small sample sizes across both studies (approximately 90 and 160 participants). Although these are relatively large sample sizes compared to most ERP studies, they are small by the standards of individual differences studies (e.g., Schonbrodt & Perugini, 2013). Data collection of ERP and neurological data is more labor-intensive and takes significantly more time than data collection using questionnaires. This is clearly a significant obstacle in conducting sound psychometric analyses of the ERPs that require large sample sizes. Nonetheless, collecting samples of 250 participants or larger when using ERP as individual difference indicators need to be considered seriously in the future. In sum, inconsistent findings and the lack of specificity between the ERN and personality may indicate that the relations are small and that they are sensitive to measures, sample characteristics, sample size, and tasks.

There are also other possibilities that our results could reflect. First, the ERN and personality traits may have more complicated relations than a simple linear correlation. For example, the ERN may be related to an interaction of traits (Hill, Samuel, & Foti, 2016). Alternatively, these small correlations could indicate that the two methods (self-report and ERP) are assessing different, but related constructs that are useful for different outcomes or in combination. For example, research shows that the ERN, trait fearfulness, and contextual stressors interacted to predict increased internalizing symptom levels in children (Meyer et al., 2017). Perhaps examining the convergence (as in correlation) of self-report and ERP indicators is not the most useful approach. Although such analyses will require even larger sample sizes than examining zero-order correlations, they may be necessary to elucidate the possible complex relations between the ERN and personality traits.

Another possible approach is to combine the ERP, self-reported traits, and other individual difference indicators to identify a shared latent construct, an approach proposed as the psychoneurometric operationalization (Patrick et al., 2013). This approach reconceptualizes the constructs as a combination of multiple methods, including ERPs, self-reports, and behavioral measures, in one big factor model to partition method variance. Although this approach requires a battery of assessment to extract the multi-modal construct in addition to a large sample size, this is an approach designed to integrate multiple methods. Of course, these are not an exhaustive list of possibilities. Rather, we hope this provides some points to consider how ERP and personality research can be integrated in the future.

### 4.1 Limitations

Several limitations should be noted. First, as already have been discussed, the sample size of 93 limits the stability of the correlation estimates. At the same time, this study used a sample size that is relatively large for an ERP study, which has a strong history in experimental designs examining *within-subject* contrasts. Although the likelihood of replication of the exact meaningful (i.e., *r* > .10) ERN and personality relation pattern found in this manuscript is limited due to sample size, we hope our manuscript provides a starting point and stimulates future rigorous psychometric studies. Second, the personality profile similarities were calculated using only five correlations (between the ERN and personality domains) that were small effect sizes. Though we think this analysis provides some insight into understanding the task similarities and differences, they should be interpreted with care. In this manuscript, we focused on the domains to correlate estimated latent scores of both the ERN and personality traits. Using longer self-report measures that allow for the testing and calculation of latent facet scores might aid in examining more stable personality profile similarities across tasks (e.g., 30 facets compared to five domains). Third, factor analysis results and factor scores are influenced by the tasks used. We used the Flanker task and stimuli that we thought were relevant to personality and psychopathology traits. However, this is not an exhaustive set of tasks or stimuli to elicit the ERN. If different tasks or stimuli are used and similar analyses are conducted, a different result (especially for General ERN) may emerge. Replication using different tasks will further inform the nature of the hierarchical structure of the ERN and its relations to personality traits.

### 4.2 Future direction

The ERN was investigated in this manuscript but many more ERPs have been discovered (e.g., Luck, 2014) and their relations to individual differences (e.g., personality, psychopathology) have been investigated without rigorous psychometric examinations. This study provided support for the assumption of unidimensionality and traditional calculation of means, at least for some ERN amplitudes. However, we also demonstrated that psychometric analyses of the ERPs are needed to test such assumptions. One important takeaway from this project is that variations across tasks can lead to variations in the ERN amplitudes elicited. How, exactly, they differ remains unclear. Nonetheless, task choice is likely an important factor in modeling the ERN and we caution against the traditional assumption of the ERN as a unitary construct (Riesel, et al. 2013). This project also confirms the small effect sizes of relations between the ERN and personality traits, likely due to strong method effects (Patrick et al., 2013). Further, the relations between the ERN and personality traits could be nonlinear and require more complicated analyses that require even larger sample sizes.

We continue to believe that the ERN is a promising trait indicator despite the lack of clear relations in this study. With that in mind, we offer six recommendations for future research examining the link between the ERN and personality measures. First and foremost, although collecting ERP data is labor-intensive and time-consuming, collecting large samples (e.g., *N* > 250) is recommended for sufficiently stable correlations. We hope that this project will urge other researchers to collect large sample sizes when examining the likely small effects between ERPs and individual difference indicators (e.g., personality, psychopathology). Second, we recommend conducting studies dedicated to examining the psychometric properties of the ERPs, as was done in this manuscript. This requires a commitment by the researcher to collect larger sample size than a typical ERP study and choosing a set of tasks for a singular purpose. This is the approach used when creating new personality measures and could be applied to establish the properties as well as the appropriateness of ERPs as individual difference indicators. Third, we recommend using omnibus trait measures, such as the FFM, to examine the convergent *and discriminant* validity of the specific relations between ERP and personality traits (Campbell & Fiske, 1959), rather than focusing on a specific trait that may be prone to Type I error. Fourth, we recommend collecting a battery of non-questionnaire-based measures of individual differences, ideally, measures that theoretically capture different constructs (e.g., reward processing, attention). This would allow the researcher to assess the validity of each indicator as well as the possibility of combining these measures to assess a latent construct underlying all methodology (Patrick et al., 2013). Combining the last two points would allow for a rigorous multi-trait multi-method approach to assessing construct validity (Campbell & Fiske, 1959). Fifth, experimenting with different modifications to the tasks and stimuli may enrich the field of ERPs and allow for more focused psychometric and personality research. In this study, we focused on social, unpleasant, and pleasant contexts using specific sets of stimuli. Changing the modality of eliciting the context (e.g., mood induction instead of using different stimuli) or switching the specific stimuli (e.g., erotic pictures to food pictures in the Pleasant task) could clarify the roles of the specific tasks and stimuli. Finally, preregistration is recommended for future research examining the relations between ERPs and personality, especially with the high likelihood of small observed effect sizes (e.g., Nosek, Ebersole, DeHaven, & Mellor, 2018). Particularly, Registered Reports (e.g., Nosek & Lakens, 2014) provides a promising avenue for conducting research with all of our recommendations, given they require a commitment of valuable resources (e.g., time, labor) by the researcher. In this model, studies will be judged by their design prior to data collection, rather than by their findings. This will increase the incentive to collect large data and variables needed for psychometric analyses.

### 4.3 Summary

The current manuscript tested the assumptions of ERN and examined its hierarchical structure as well as the uniqueness of the ERN amplitudes elicited from different tasks. This project was specifically designed to investigate the psychometric properties of the ERN elicited from four tasks and their relations to personality dimensions. The unidimensionality of the ERN was supported for three out of four tasks and a slight modification was required for the Pleasant task to achieve unidimensionality. All ERN amplitudes – across tasks – share a common variance but also have unique task-specific variances. This study found that some assumptions of the ERN were supported while some were not, demonstrating the utility and importance of using psychometric techniques to examine and establish the ERN as an indicator of individual differences. The relations of the ERN elicited from different tasks to personality traits were small and remain unclear. However, more importantly, the tasks do seem to make a difference. The ERN continues to be a promising neurological indicator of individual differences and exhibit a dimensional hierarchical structure, similar to that found using self-reported measures. Further research dedicated to examining the psychometric properties of the ERN and the roles of the task could aid in integrating these methodologies into personality and psychopathology research.

## Footnotes

1 Given the high loadings of the picture-based tasks, a second-order CFA testing a model with one latent task for these tasks was conducted. Specifically, the five units from the Arrow task loaded onto one Arrow latent task construct and the remaining 14 units loaded onto another Picture latent task construct. Both task constructs, in turn, loaded onto a general latent factor. This model fit well (Table 1, last row) but with higher AIC and BIC. Further inspection of the model also indicated a negative variance of the Picture latent task construct, indicating problems with the model. Therefore, the second-order model with each task having its own latent task construct was retained.

## Supplementary material

For supplementary material accompanying this paper visit https://doi.org/10.1017/pen.2020.15. click here to view supplementary material
