# Supplementary material for: The hierarchical structure of error-related negativities elicited from affective and social stimuli and their relations to personality traits
Source: Personal Neurosci. 2021 Jan 5;3:e15. doi: 10.1017/pen.2020.15 (PMC7808877; doi:10.1017/pen.2020.15)
Supplement: Supplementary file 1 [file S2513988620000152sup001.docx]

Supplemental Table 1. *Within-Subjects Outlier Removal Procedure Results*.

|  | Arrow | Social | Unpleasant | Pleasant |
| --- | --- | --- | --- | --- |
| Number of Total Trials Removed | 98 | 127 | 77 | 91 |
| Number of Participants Affected | 58 | 71 | 49 | 56 |
| Average Trials per Participant Removed | 1.69 | 1.79 | 1.57 | 1.63 |
| Maximum Number of Trials Removed from a Single Participant | 5 | 7 | 4 | 5 |

Supplemental Table 2. *Descriptive Statistics of the Errors Made.*

| Task | Unit | No Errors | M | SD | Min | Max |
| --- | --- | --- | --- | --- | --- | --- |
| Arrow (N = 91) | 1 | 3 | 4.68 | 3.04 | 1 | 17 |
|  | 2 | 1 | 6.24 | 3.26 | 1 | 17 |
|  | 3 | 0 | 6.52 | 3.21 | 1 | 15 |
|  | 4 | 3 | 7.42 | 3.68 | 2 | 19 |
|  | 5 | 1 | 7.44 | 3.29 | 1 | 16 |
|  | All | 0 | 31.76 | 12.06 | 5 | 65 |
| Social (N = 93) | 1 | 0 | 7.52 | 3.02 | 2 | 15 |
|  | 2 | 0 | 8.26 | 3.11 | 2 | 19 |
|  | 3 | 1 | 8.13 | 2.79 | 3 | 16 |
|  | 4 | 0 | 8.60 | 3.39 | 2 | 18 |
|  | 5 | 0 | 8.48 | 3.43 | 1 | 18 |
|  | All | 0 | 40.90 | 11.56 | 10 | 73 |
| Unpleasant (N = 91) | 1 | 0 | 8.75 | 4.80 | 1 | 24 |
|  | 2 | 1 | 9.38 | 3.88 | 1 | 17 |
|  | 3 | 0 | 8.88 | 3.49 | 1 | 17 |
|  | 4 | 0 | 9.16 | 3.89 | 1 | 18 |
|  | 5 | 1 | 8.66 | 3.28 | 1 | 17 |
|  | All | 0 | 44.63 | 15.33 | 7 | 70 |
| Pleasant (N = 89) | 1 | 1 | 7.31 | 3.88 | 1 | 19 |
|  | 2 | 1 | 8.01 | 3.41 | 1 | 15 |
|  | 3 | 1 | 8.56 | 3.57 | 1 | 18 |
|  | 4 | 0 | 8.92 | 4.18 | 1 | 19 |
|  | 5 | 0 | 8.45 | 3.39 | 2 | 16 |
|  | All | 0 | 40.98 | 13.25 | 7 | 71 |

*Notes*. N = Sample Size; No Errors = Number of participants who did not make any error in the Unit. M = Mean; SD = Standard deviation; Min = Minimum value; Max = Maximum value.

Supplemental Table 3. *Descriptive Statistics of the Error-Related Negativity Variables.*

| Task | Unit | N | M (µV) | SD | Min | Max | Skew | Kurtosis |
| --- | --- | --- | --- | --- | --- | --- | --- | --- |
| Arrow | 1 | 88 | -7.02 | 7.07 | -30.26 | 8.71 | -0.30 | 0.80 |
|  | 2 | 90 | -6.57 | 7.44 | -28.43 | 12.80 | -0.04 | 0.39 |
|  | 3 | 91 | -5.53 | 6.98 | -27.46 | 15.97 | -0.12 | 0.82 |
|  | 4 | 88 | -6.02 | 5.99 | -21.05 | 8.17 | -0.25 | 0.34 |
|  | 5 | 89 | -5.75 | 6.29 | -28.00 | 12.54 | -0.27 | 1.19 |
|  | All | 91 | -5.88 | 4.96 | -21.68 | 5.78 | -0.64 | 0.97 |
| Social | 1 | 91 | -6.12 | 5.15 | -24.70 | 5.66 | -0.76 | 1.10 |
|  | 2 | 93 | -5.38 | 5.59 | -27.91 | 5.83 | -0.77 | 1.48 |
|  | 3 | 90 | -5.80 | 4.63 | -19.58 | 9.21 | -0.30 | 1.07 |
|  | 4 | 91 | -5.53 | 5.24 | -16.89 | 7.16 | 0.08 | -0.33 |
|  | 5 | 91 | -5.70 | 5.19 | -21.28 | 8.58 | -0.27 | 0.53 |
|  | All | 92 | -5.60 | 3.85 | -21.16 | 3.96 | -0.79 | 1.91 |
| Unpleasant | 1 | 90 | -7.39 | 5.07 | -19.29 | 4.12 | 0.15 | -0.48 |
|  | 2 | 88 | -7.65 | 6.03 | -26.01 | 8.24 | -0.43 | 0.51 |
|  | 3 | 89 | -6.63 | 6.01 | -21.71 | 10.49 | -0.28 | -0.05 |
|  | 4 | 89 | -6.50 | 6.30 | -25.46 | 18.56 | 0.94 | 4.16 |
|  | 5 | 87 | -8.15 | 5.12 | -19.97 | 5.48 | 0.05 | -0.29 |
|  | All | 89 | -7.39 | 3.85 | -16.85 | 2.89 | -0.31 | 0.22 |
| Pleasant | 1 | 87 | -6.79 | 6.03 | -24.48 | 4.47 | -0.67 | 0.29 |
|  | 2 | 87 | -8.75 | 5.29 | -35.02 | 3.11 | -1.37 | 5.54 |
|  | 3 | 85 | -8.91 | 6.77 | -33.75 | 3.04 | -1.16 | 1.99 |
|  | 4 | 88 | -8.58 | 6.38 | -24.25 | 7.99 | -0.28 | 0.00 |
|  | 5 | 87 | -7.16 | 5.37 | -21.70 | 7.07 | -0.20 | -0.05 |
|  | All | 88 | -7.95 | 4.06 | -22.97 | -0.50 | -0.79 | 0.95 |

*Notes*. N = Sample Size; M = Mean; µV = microvolts; SD = Standard deviation; Min = Minimum value; Max = Maximum value.

Supplemental Table 4. *Correlations of Units and Grand Averages Within Each Task.*

| Arrow |  | Unit 1 | Unit 2 | Unit 3 | Unit 4 | Unit 5 |
| --- | --- | --- | --- | --- | --- | --- |
|  | Unit 2 | 0.41 |  |  |  |  |
|  | Unit 3 | 0.45 | 0.47 |  |  |  |
|  | Unit 4 | 0.52 | 0.42 | 0.56 |  |  |
|  | Unit 5 | 0.40 | 0.31 | 0.41 | 0.45 |  |
|  | Grand Average | 0.72 | 0.69 | 0.72 | 0.80 | 0.70 |
| Social |  | Unit 1 | Unit 2 | Unit 3 | Unit 4 | Unit 5 |
|  | Unit 2 | 0.53 |  |  |  |  |
|  | Unit 3 | 0.25 | 0.35 |  |  |  |
|  | Unit 4 | 0.27 | 0.36 | 0.40 |  |  |
|  | Unit 5 | 0.26 | 0.33 | 0.30 | 0.24 |  |
|  | Grand Average | 0.69 | 0.79 | 0.67 | 0.68 | 0.59 |
| Unpleasant |  | Unit 1 | Unit 2 | Unit 3 | Unit 4 | Unit 5 |
|  | Unit 2 | 0.42 |  |  |  |  |
|  | Unit 3 | 0.22 | 0.37 |  |  |  |
|  | Unit 4 | 0.30 | 0.32 | 0.34 |  |  |
|  | Unit 5 | 0.22 | 0.48 | 0.32 | 0.36 |  |
|  | Grand Average | 0.56 | 0.78 | 0.67 | 0.65 | 0.67 |
| Pleasant |  | Unit 1 | Unit 2 | Unit 3 | Unit 4 | Unit 5 |
|  | Unit 2 | 0.43 |  |  |  |  |
|  | Unit 3 | 0.16 | 0.35 |  |  |  |
|  | Unit 4 | 0.39 | 0.29 | 0.39 |  |  |
|  | Unit 5 | 0.17 | 0.25 | 0.24 | 0.53 |  |
|  | Grand Average | 0.61 | 0.68 | 0.62 | 0.78 | 0.69 |

Supplemental Table 5. *Standardized Factor Loadings of Units Onto Latent Task Construct for Each Task.*

| Unit | Arrow | Social | Unpleasant | Pleasant (2) | Pleasant (4) |
| --- | --- | --- | --- | --- | --- |
| 1 | 0.648 | 0.642 | 0.506 | 0.402 | 0.515 |
| 2 | 0.594 | 0.742 | 0.739 |  | 0.803 |
| 3 | 0.728 | 0.598 | 0.531 | 0.427 | 0.508 |
| 4 | 0.752 | 0.600 | 0.549 | 0.960 |  |
| 5 | 0.589 | 0.463 | 0.646 | 0.567 | 0.411 |

Supplemental Table 6. *Descriptive Statistics of the International Personality Item Pool-NEO and Personality Inventory for DSM-5 Traits.*

| Model | Domain | Facet | N | M | SD | Min. | Max. | Skew | Kurtosis |
| --- | --- | --- | --- | --- | --- | --- | --- | --- | --- |
| IPIP-NEO | Neuroticism | N1 | 93 | 3.61 | 0.84 | 1.00 | 5.00 | -0.84 | 0.92 |
|  |  | N2 | 93 | 2.79 | 0.97 | 1.00 | 5.00 | -0.03 | -0.56 |
|  |  | N3 | 93 | 2.88 | 1.07 | 1.00 | 5.00 | 0.14 | -0.91 |
|  |  | N4 | 93 | 2.86 | 0.66 | 1.25 | 4.25 | -0.16 | -0.44 |
|  |  | N5 | 93 | 3.19 | 0.76 | 1.25 | 5.00 | 0.02 | -0.55 |
|  |  | N6 | 93 | 2.74 | 0.70 | 1.00 | 4.25 | -0.15 | -0.76 |
|  | Extraversion | E1 | 93 | 3.58 | 0.82 | 1.00 | 5.00 | -0.48 | 0.10 |
|  |  | E2 | 93 | 2.97 | 1.14 | 1.00 | 5.00 | -0.13 | -1.24 |
|  |  | E3 | 93 | 3.34 | 0.86 | 1.25 | 5.00 | -0.07 | -0.72 |
|  |  | E4 | 93 | 3.35 | 0.70 | 1.50 | 5.00 | -0.13 | -0.03 |
|  |  | E5 | 93 | 3.66 | 0.75 | 1.75 | 5.00 | -0.45 | -0.18 |
|  |  | E6 | 93 | 3.79 | 0.78 | 1.75 | 5.00 | -0.54 | -0.41 |
|  | Openness to Experience | O1 | 93 | 3.89 | 0.86 | 1.50 | 5.00 | -0.59 | -0.36 |
|  |  | O2 | 93 | 3.67 | 0.85 | 1.75 | 5.00 | -0.45 | -0.50 |
|  |  | O3 | 93 | 3.70 | 0.87 | 1.25 | 5.00 | -0.31 | -0.52 |
|  |  | O4 | 93 | 2.88 | 0.83 | 1.25 | 5.00 | 0.34 | -0.12 |
|  |  | O5 | 93 | 3.70 | 0.85 | 2.00 | 5.00 | -0.15 | -1.00 |
|  |  | O6 | 93 | 2.88 | 0.97 | 1.00 | 5.00 | 0.09 | -0.38 |
|  | Agreeableness | A1 | 93 | 3.44 | 0.78 | 1.75 | 4.75 | -0.34 | -0.91 |
|  |  | A2 | 93 | 3.68 | 0.60 | 2.25 | 5.00 | -0.08 | -0.43 |
|  |  | A3 | 93 | 4.34 | 0.50 | 3.00 | 5.00 | -0.64 | -0.08 |
|  |  | A4 | 93 | 4.00 | 0.79 | 2.00 | 5.00 | -0.49 | -0.69 |
|  |  | A5 | 93 | 3.43 | 0.88 | 1.50 | 5.00 | 0.00 | -0.62 |
|  |  | A6 | 93 | 4.00 | 0.66 | 2.25 | 5.00 | -0.25 | -0.64 |
|  | Conscientiousness | C1 | 93 | 3.82 | 0.55 | 2.25 | 5.00 | -0.44 | 0.30 |
|  |  | C2 | 93 | 3.35 | 0.86 | 1.25 | 5.00 | -0.19 | -0.61 |
|  |  | C3 | 93 | 4.15 | 0.54 | 2.50 | 5.00 | -0.54 | 0.12 |
|  |  | C4 | 93 | 3.92 | 0.70 | 1.25 | 5.00 | -0.74 | 1.05 |
|  |  | C5 | 93 | 2.74 | 0.98 | 1.00 | 4.75 | -0.04 | -1.08 |
|  |  | C6 | 93 | 3.06 | 0.93 | 1.00 | 5.00 | -0.31 | -0.44 |
| Model | Domain | Facet | N | M | SD | Min. | Max. | Skew | Kurtosis |
| PID-5 | Negative Affectivity | Anxiousness | 93 | 1.91 | 0.72 | 0.00 | 3.00 | -0.54 | -0.37 |
|  |  | Emotional Lability | 93 | 1.22 | 0.78 | 0.00 | 2.75 | 0.10 | -0.89 |
|  |  | Hostility | 93 | 0.84 | 0.75 | 0.00 | 2.75 | 0.72 | -0.32 |
|  |  | Perseveration | 93 | 1.32 | 0.62 | 0.00 | 3.00 | 0.24 | -0.64 |
|  |  | Restricted Affectivity | 93 | 4.03 | 0.79 | 2.00 | 5.00 | -0.69 | -0.37 |
|  |  | Separation Insecurity | 93 | 1.56 | 0.74 | 0.00 | 3.00 | -0.11 | -0.54 |
|  |  | Submissiveness | 93 | 1.63 | 0.59 | 0.25 | 3.00 | -0.14 | -0.34 |
|  | Detachment | Anhedonia | 93 | 0.80 | 0.69 | 0.00 | 2.75 | 0.81 | 0.04 |
|  |  | Depressivity | 93 | 0.61 | 0.71 | 0.00 | 2.75 | 1.08 | 0.13 |
|  |  | Intimacy Avoidance | 93 | 0.74 | 0.67 | 0.00 | 3.00 | 0.95 | 0.60 |
|  |  | Suspiciousness | 93 | 0.85 | 0.55 | 0.00 | 2.25 | 0.54 | -0.37 |
|  |  | Withdrawal | 93 | 0.80 | 0.64 | 0.00 | 2.50 | 0.64 | -0.22 |
|  | Psychoticism | Eccentricity | 93 | 1.51 | 0.92 | 0.00 | 3.00 | -0.09 | -1.12 |
|  |  | Perceptual Dysregulation | 93 | 0.61 | 0.59 | 0.00 | 2.25 | 0.74 | -0.25 |
|  |  | Unusual Beliefs Experiences | 93 | 0.88 | 0.70 | 0.00 | 2.50 | 0.30 | -1.01 |
|  | Antagonism | Attention Seeking | 93 | 1.29 | 0.83 | 0.00 | 3.00 | 0.18 | -0.96 |
|  |  | Callousness | 93 | 0.27 | 0.43 | 0.00 | 1.75 | 1.72 | 2.28 |
|  |  | Deceitfulness | 93 | 0.73 | 0.56 | 0.00 | 2.25 | 0.66 | -0.38 |
|  |  | Grandiosity | 93 | 0.35 | 0.48 | 0.00 | 2.25 | 1.54 | 2.09 |
|  |  | Manipulativeness | 93 | 0.86 | 0.63 | 0.00 | 2.50 | 0.69 | -0.29 |
|  | Disinhibition | Distractibility | 93 | 1.79 | 0.74 | 0.25 | 3.00 | -0.04 | -1.00 |
|  |  | Impulsivity | 93 | 1.09 | 0.74 | 0.00 | 3.00 | 0.57 | -0.35 |
|  |  | Irresponsibility | 93 | 0.48 | 0.51 | 0.00 | 2.75 | 1.45 | 3.23 |
|  |  | Rigid Perfectionism | 93 | 3.77 | 0.72 | 2.25 | 5.00 | -0.09 | -1.00 |
|  |  | Risk Taking | 93 | 0.98 | 0.76 | 0.00 | 3.00 | 0.66 | -0.24 |

*Notes*. N = Sample Size; M = Mean; SD = Standard deviation; Min = Minimum value; Max = Maximum value; IPIP-NEO = International Personality Item Pool-NEO; PID-5 = Personality Inventory for DSM-5.

Supplemental Table 7. *Correlations of Estimated Latent Five-Factor Model and Personality Inventory for DSM-5 Domain Traits.*

| Measure |  | IPIP-NEO | | | | | PID-5 | | | |
| --- | --- | --- | --- | --- | --- | --- | --- | --- | --- | --- |
|  | Domain (Dropped Facet) | N | E (3) | O (4) | A (3 & 5) | C (5) | NA (Res.) | DE (Wit.) | PS | AN (Gra.) |
| IPIP-NEO | E (3) | -0.35 |  |  |  |  |  |  |  |  |
|  | O (4) | 0.02 | 0.11 |  |  |  |  |  |  |  |
|  | A (3 & 5) | -0.14 | 0.01 | 0.28 |  |  |  |  |  |  |
|  | C (5) | -0.23 | 0.29 | 0.16 | 0.36 |  |  |  |  |  |
| PID-5 | NA (Res.) | 0.74 | -0.20 | 0.00 | -0.23 | -0.38 |  |  |  |  |
|  | DE (Wit.) | **0.52** | **-0.53** | 0.04 | -0.18 | -0.46 | 0.49 |  |  |  |
|  | Psychoticism | 0.05 | 0.01 | 0.24 | -0.27 | -0.25 | 0.18 | 0.22 |  |  |
|  | AN (Gra.) | 0.18 | -0.03 | -0.14 | **-0.68** | -0.46 | 0.35 | 0.22 | 0.45 |  |
|  | DI (Dis.) | -0.13 | 0.21 | 0.15 | -0.23 | -0.35 | 0.03 | 0.12 | **0.60** | 0.40 |

*Notes*. IPIP-NEO = International Personality Item Pool-NEO; PID-5 = Personality Inventory for DSM-5; N = Neuroticism; E = Extraversion; O = Openness to Experience; A = Agreeableness; C = Conscientiousness; NA = Negative Affectivity; Res. = Restricted Affectivity; DE = Detachment; Wit. = Withdrawal; AN = Antagonism; Gra. = Grandiosity; PS = Psychoticism; DI = Disinhibition; Dis = Distractibility; Underline = |*r*| > .30; Bold = |*r*| > .50.

Supplemental Table 8. *Correlations of Estimated Latent Task Error-Related Negativity Amplitudes from Second-Order Confirmatory Factor Analyses with Estimated Latent Five-Factor Model and Personality Inventory for DSM-5 Domain Traits.*

|  |  | Second-Order CFA | | | |
| --- | --- | --- | --- | --- | --- |
| Model | Domain | Arrow | Social | Unpleasant | Pleasant |
| IPIP-NEO | Neuroticism | **0.06** | 0.01 | **-0.05** | -0.03 |
|  | Extraversion | 0.03 | **-0.06** | 0.00 | **-0.02** |
|  | Openness | -0.11 | -0.03 | -0.02 | 0.01 |
|  | Agreeableness | -0.14 | **-0.06** | -0.16 | -0.06 |
|  | Conscientiousness | **-0.10** | -0.03 | -0.01 | -0.06 |
| PID-5 | Negative Affectivity | **0.01** | -0.07 | **-0.09** | -0.10 |
|  | Detachment (-E) | -0.15 | **-0.03** | -0.04 | **-0.02** |
|  | Psychoticism | 0.03 | 0.05 | 0.13 | 0.09 |
|  | Antagonism (-A) | 0.10 | **0.02** | 0.11 | 0.05 |
|  | Disinhibition (-C) | **0.09** | -0.01 | 0.04 | 0.04 |

*Notes*. CFA = Confirmatory factor analysis; IPIP-NEO = International Personality Item Pool-NEO; PID-5 = Personality Inventory for DSM-5; -E = Opposite of Extraversion; -A = Opposite of Agreeableness; -C = Opposite of Conscientiousness; Bold = Hypothesized relations; Underline = |*r*| > .10.

Supplemental Table 9. *Personality Profile Similarities of Estimated Latent Task Error-Related Negativity Amplitudes from Second-Order Confirmatory Factor Analyses.*

|  |  |  | Second-Order CFA | | | |  |  | Second-Order CFA | | | |
| --- | --- | --- | --- | --- | --- | --- | --- | --- | --- | --- | --- | --- |
|  |  |  | Arrow | Social | Unpleasant | Pleasant |  |  | Arrow | Social | Unpleasant | Pleasant |
| Using IPIP-NEO Domains | Single Task CFA | Arrow | **1.00** | 0.43 | 0.37 | 0.17 | Second-Order CFA | Social | 0.40 |  |  |  |
|  |  | Social | -0.08 | **0.79** | -0.27 | -0.14 |  | Unpleasant | 0.44 | 0.28 |  |  |
|  |  | Unpleasant | 0.29 | 0.12 | **0.98** | 0.48 |  | Pleasant | 0.20 | 0.28 | 0.48 |  |
|  |  | Pleasant | -0.47 | 0.01 | 0.13 | **0.77** |  | General | **0.68** | **0.66** | **0.79** | **0.73** |
| Using PID-5 Domains |  | Arrow | **0.97** | 0.24 | 0.38 | 0.23 |  | Social | 0.42 |  |  |  |
|  |  | Social | -0.42 | 0.47 | 0.35 | 0.31 |  | Unpleasant | **0.55** | **0.99** |  |  |
|  |  | Unpleasant | **0.56** | **0.97** | **0.99** | **0.97** |  | Pleasant | 0.43 | **0.97** | **0.96** |  |
|  |  | Pleasant | -0.20 | **0.73** | **0.63** | **0.77** |  | General | **0.58** | **0.98** | **0.99** | **0.98** |

*Notes*. CFA = Confirmatory factor analysis; IPIP-NEO = International Personality Item Pool-NEO; PID-5 = Personality Inventory for DSM-5; Underline = |*r*| > .30; Bold = |*r*| > .50; Top four rows used correlations with IPIP-NEO domains to calculate the similarity. Bottom four rows used correlations with PID-5 domains to calculate the similarity.


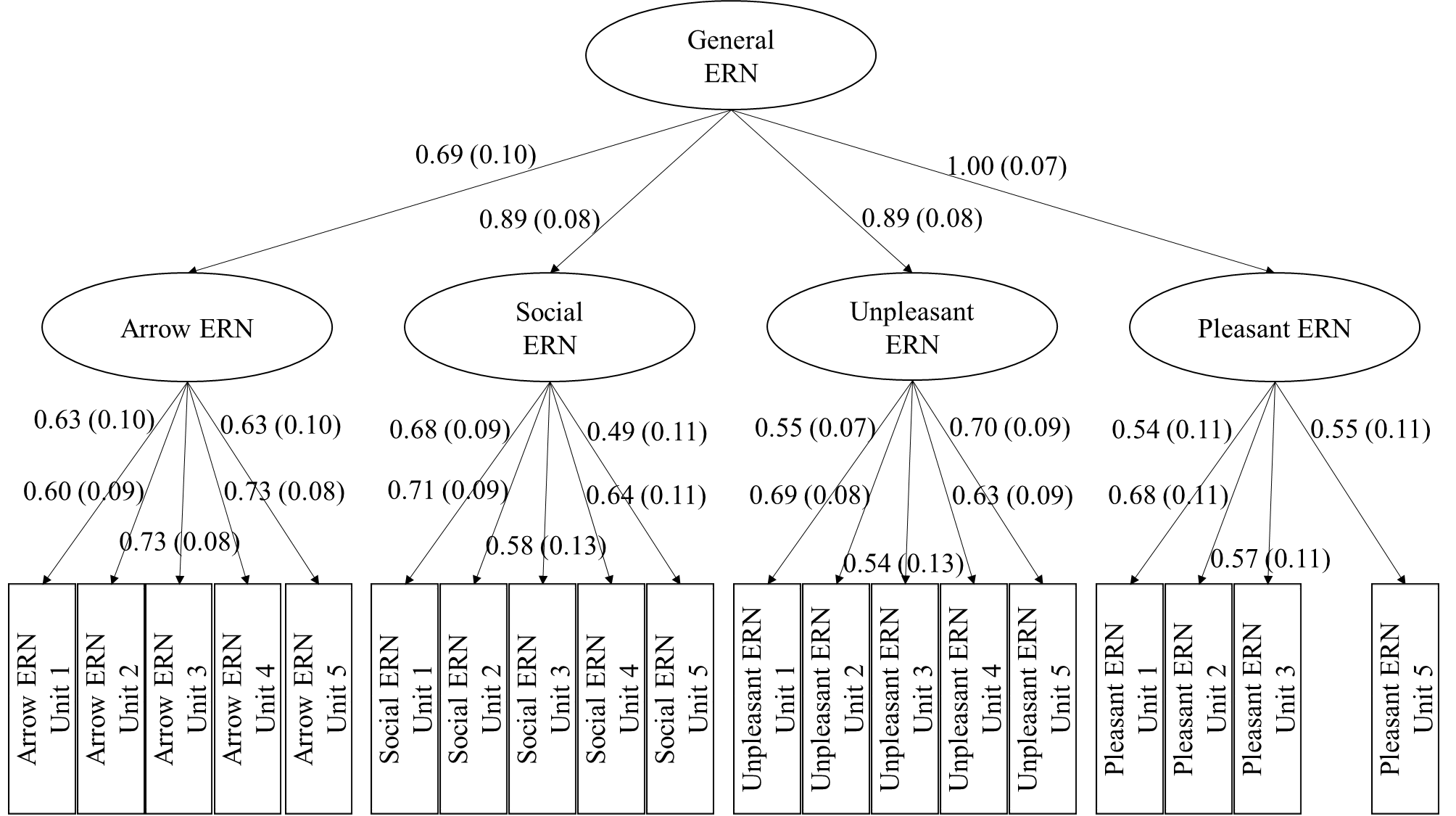


Supplemental Figure 1. *The final second-order confirmatory factor analysis model with standardized factor loadings (Standard errors in parentheses).*


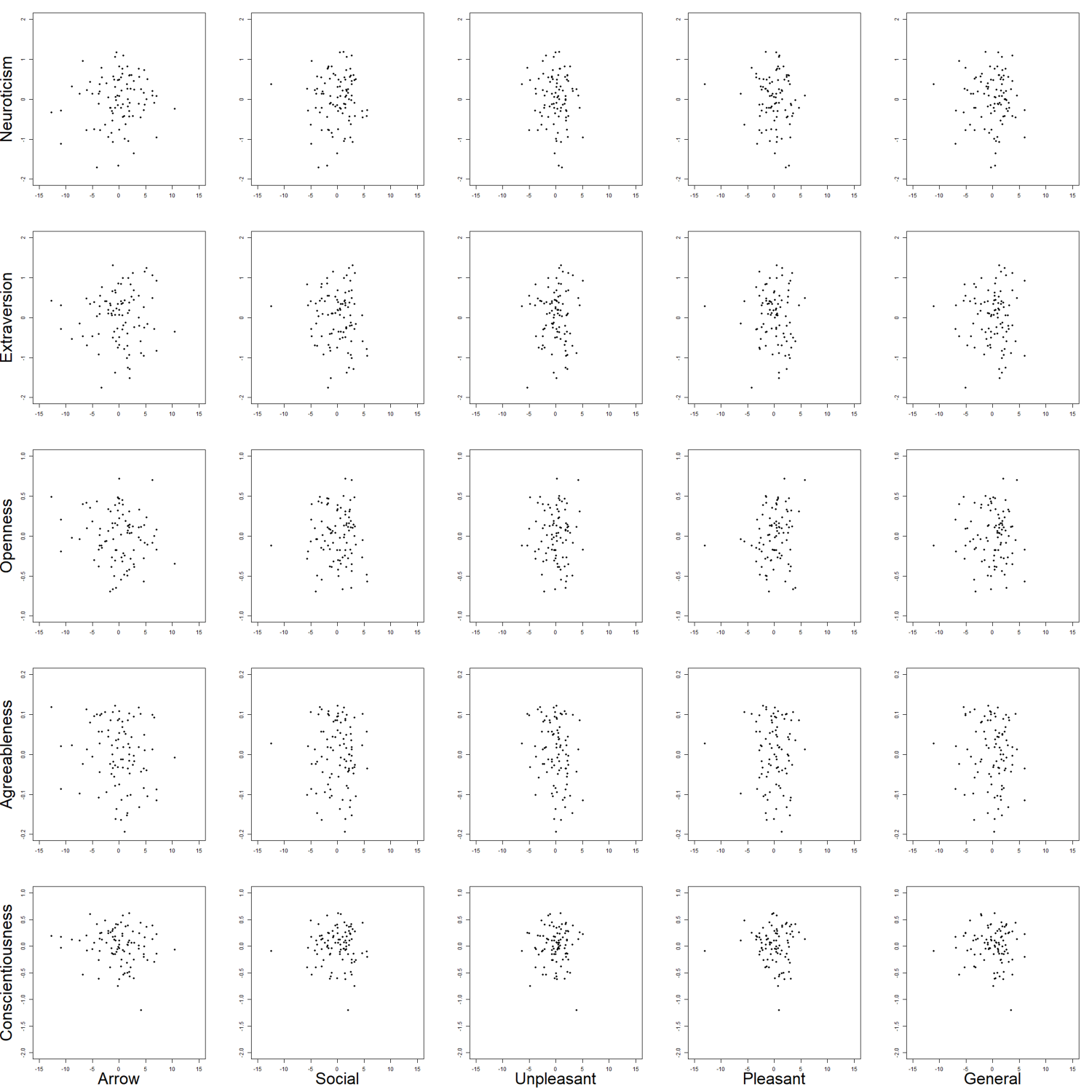


Supplemental Figure 2. Scatter Plot Between Estimated Latent Error-Related Negativity Amplitudes with Estimated General International Personality Item Pool-NEO Domain Traits. See Table 3 for the correlation values.


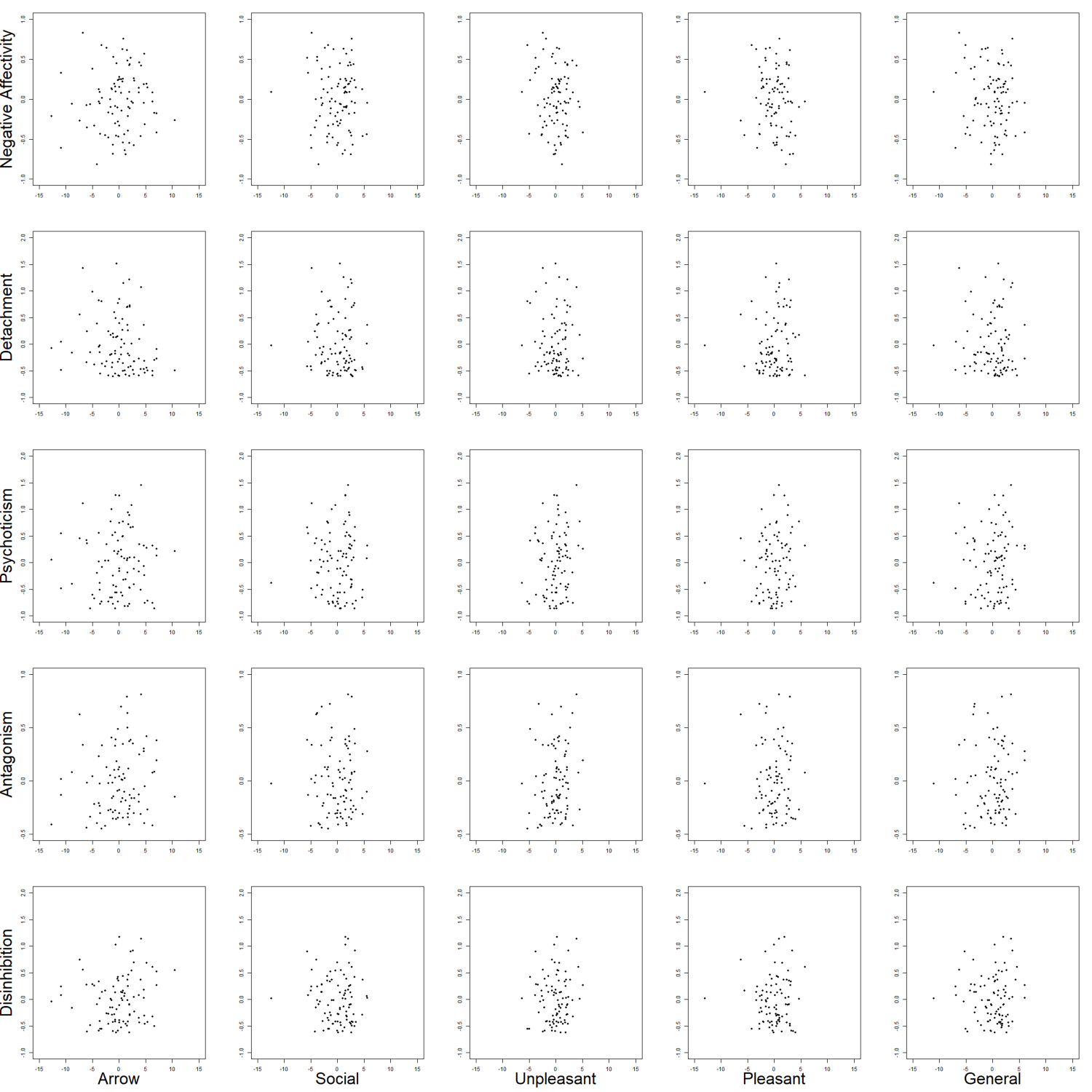


Supplemental Figure 3. Scatter Plot Between Estimated Latent Error-Related Negativity Amplitudes with Estimated Maladaptive Five-Factor Model Domain Traits. See Table 3 for the correlation values.
